# Association between breastfeeding and periodontitis in Korean women using Korea National Health and Nutrition Examination Survey (KNHANES): a cross-sectional study

**DOI:** 10.1186/s12903-023-03213-6

**Published:** 2023-07-17

**Authors:** Zi-Lan Wang, Seung-Hee Ryu, Kwang-Hak Bae, Seon-Jip Kim, Hyun-Jae Cho

**Affiliations:** 1 Department of Preventive Dentistry & Public Oral Health, School of Dentistry and Dental Research Institute, Seoul National University, Daehak-Ro, Jongro-Gu, Seoul, South Korea; 2 Seoul SUN Dental Hospital, Paju-Si, Gyeonggi-Do, Seoul, South Korea

**Keywords:** Breastfeeding, Periodontitis, Korean women

## Abstract

**Objectives:**

The effect of breastfeeding on periodontal disease in women remains unclear. This cross-sectional study used data from the Korean National Health and Nutrition Examination Survey to explore the association between breastfeeding and periodontitis in Korean women using data from the Korean National Health and Nutrition Examination Survey (KNHANES VII).

**Materials and methods:**

Cross-sectional data was analyzed from the KNHANES 2016–2018. The study population included 5,587 parous women aged ≥ 30 years. The outcome variable was the presence or absence of periodontitis. The explanatory variable, period of breastfeeding, was defined as “none”, “1–11 months”, and “more than 12 months”. Confounder variables (socio-educational, personal healthcare practice, and systemic medical characteristics) were adjusted for in the logistic regression analysis.

**Results:**

Approximately 60% of the participants breastfed for ≥ 12 months. In all statistical models, the prevalence of periodontitis was approximately 60% greater in women that did not breastfeed compared to women that had breastfed for 12 months or longer. When adjusted for age, statistical significance was only present in the 50–59 years age group (adjusted odds ratio [aOR], 1.678; 95% confidence interval [CIs], 1.046–2.691).

**Conclusion:**

Our study shows that women that breastfed for a relatively long duration had a lower risk of periodontitis. Therefore, breastfeeding may be beneficial for women’s periodontal health. These results are expected to be helpful in oral health education for pregnant women.

## Introduction

Periodontal disease is one of the most common public health problems, affecting more than 20%-50% of the global population [1]. It is a complex, multi-factorial disease related to dental biofilms [2]. As a chronic inflammatory disease, periodontitis can negatively affect diet, cosmetic, speech, and other aspects of life [3]. Furthermore, there is substantial evidence that periodontitis negatively affects systemic health through various biological mechanisms [4]. A review of periodontitis in women showed that decreased estrogen levels during menopause causes bone loss and leads to recurrence or exacerbation of periodontitis [5]. As women comprise most of the elderly population and the influence of hormones on certain oral diseases has been established, maintaining good oral health throughout a woman's life is an ongoing challenge [6].

It is widely known that breast milk contains various nutrients, and breastfeeding has many health benefits not only for the child but also for the mother [7, 8]. Breastfeeding can reduce maternal risks of breast cancer, ovarian cancer, diabetes, hypertension, cardiovascular disease, and postpartum depression [9–12]. The World Health Organization recommends exclusive breastfeeding for six months and continued breastfeeding for up to two years [13]. Although breastfeeding rates have increased, 60% of the mothers wish to stop breastfeeding [14]. The health effects of breastfeeding in maternal health have been widely reported, there have been few studies on breastfeeding and maternal oral health.

In South Korea, breastfeeding rates were high in the 1980s and the 1990s, and then began to decline and reached an all-time low in 2000 [15]. A recent study in South Korea showed that the breastfeeding rate increased to 81.5% between 2010 and 2018 [16]. However, compared to other Asian countries, breastfeeding rates in South Korea are lower than that in China (96.1%), Singapore (96%), and India (95.5%) [17].

The are several factors that affect breastfeeding rates [18, 19]. At the personal level, the benefits of breastfeeding are individually subjective factors that influence whether women choose to breastfeed [20]. Although there are many studies on the maternal benefits of breastfeeding in the existing research [20–23], the oral health benefits of breastfeeding are poorly reported. To the best of our knowledge, only two previous studies have reported the relationship between breastfeeding and periodontitis. Both studies found a negative association [24, 25].

While previous studies provide some possible mechanism for a negative association between breastfeeding and periodontitis, it is without considering potential confounding factors that could influence this relationship, such as diabetes, hypercholesterolemia, and hypertriglyceridemia. In addition, in the breastfeeding period classification of one study, a group less than 18 months was used as a reference and up to 73 months were classified. In another study, breastfeeding period are in years as the continuous variable and the highest period is 15 years. It can be unreasonable classification considering the current situation in which the average birth rates were less than two. By taking these factors into account in our studies, we can better understand the mechanisms underlying the association between breastfeeding and periodontitis.

There is little evidence regarding the precise mechanisms underlying the association between breastfeeding and periodontitis. Thus, the effects of breastfeeding on periodontal health remain largely unknown. Exploring the association between breastfeeding and periodontitis has both scientific and social implications. Therefore, this study aimed to evaluate the effect of breastfeeding behavior on periodontal health in Korean women, thereby providing support for oral health education for lactating women.

### Materials & methods

#### Data and participants

**Fig. 1:**
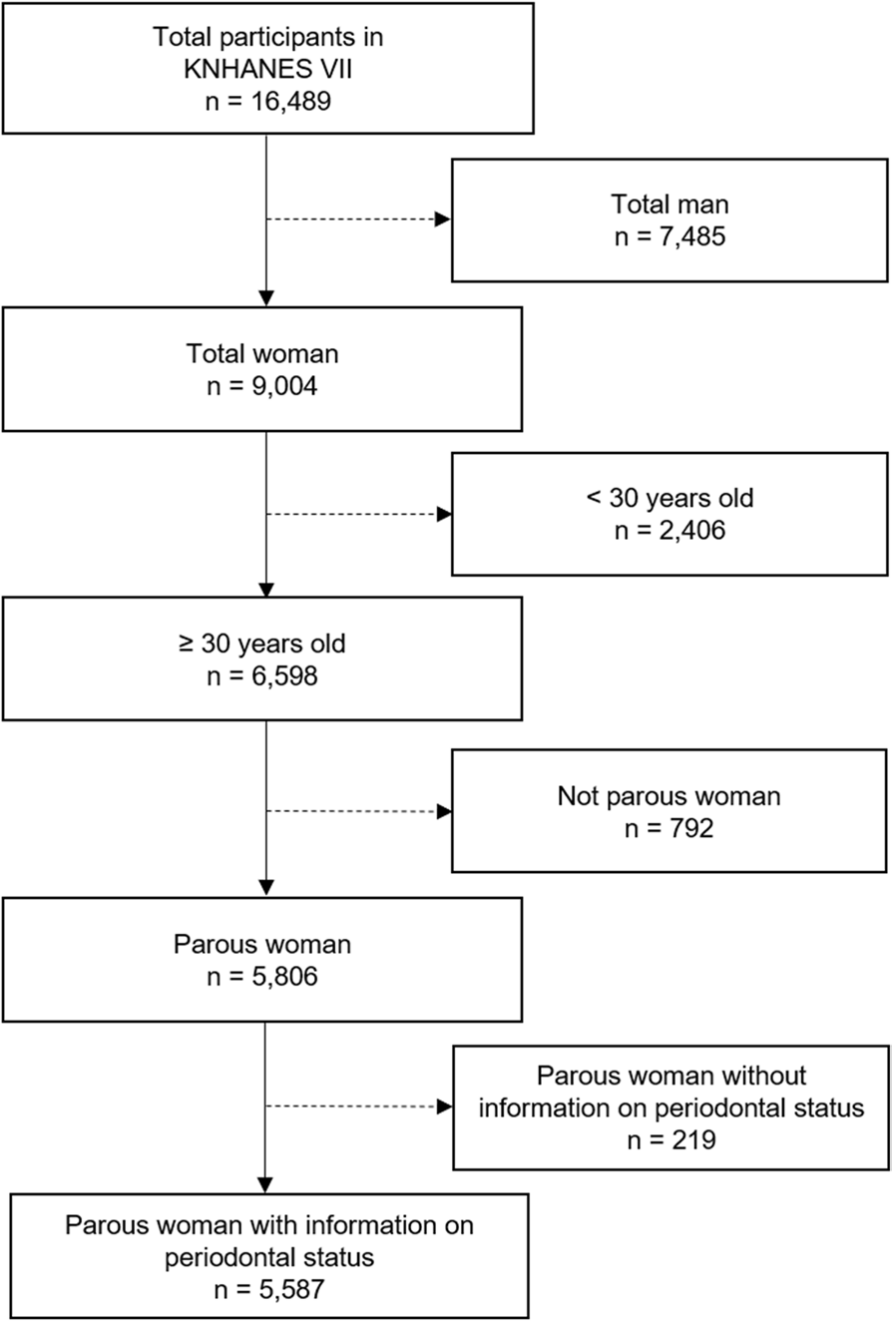
The flowchart of participants

Data from the Seventh Korea National Health and Nutrition Examination Survey (KNHANES VII-3) were used. The survey was carried out from 2016 to 2018 by the Korea Centers for Disease Control and Prevention (KCDCP) [26]. The KNHANES is a cross-sectional survey based on the national population of Korea [26]. The samples were randomly drawn from 576 distribution areas in South Korea. We included data from women over the age of 30 who gave birth and participated in the 7th KNHANES. We excluded participants with no information on periodontal disease status. All participants provided written informed consent before participation. KNHANES VII-3 was approved by the Korea Disease Control and Prevention Agency (KDCA) Research Ethics Committee on the basis of the relevant regulations. The approval number for this study was 2018–01-03-P-A. The process of recruiting is shown in Fig. 1.

### Outcome variable

The presence or absence of periodontitis was the outcome variable. Periodontal health was assessed by dentists using the Community Periodontal Index (CPI) [27]. First, reference teeth were selected from each quadrant of the left and right posterior and anterior maxillary and left and right mandibular posterior and anterior regions. The probing depth and bleeding index were measured. The following scale was used for measurement results: 0 points (perfectly healthy periodontal tissue); 1 point (bleeding periodontal tissue); 2 points (calculus formation in periodontal tissue); 3 points (superficial periodontal pocket formation (4–5 mm)); 4 points (deep periodontal pockets (≥ 6 mm)). Finally, the highest point in each quadrant was recorded and periodontitis was defined as a CPI score of 3 or 4.

### Explanatory variable

Period of breastfeeding was used as the explanatory variable and was defined as “no”, “1–11 months”, and “more than 12 months”. The classification criteria of this were established by referring to previous studies, conducting a frequency analysis, and dividing the data into three groups [24]. In the health survey of the KNHANES, there was a health interview survey about breastfeeding experience and duration of breastfeeding for women over 19 years of age. Two questions in the survey on female health assessed history of breastfeeding and breastfeeding duration.

### Covariates

Except for age, all covariates were classified into three factors: socio-educational, personal healthcare practice, and systematic medical factors [26, 28]. Socio-educational variables included household income levels (categorical: lowest, medium–low, medium–high, and highest) and educational levels (categorical: lower primary school, middle school, high school, and college or higher). Variables of personal healthcare practice included the use of dental floss (yes or no), use of interdental brushes (yes or no), daily brushing frequency (less than once, twice, and more than three times per day), and self-rated oral health (poor or good). Medical status variables included smoking status (categorical, never, past, or current), alcohol consumption (categorical, never, or current), diabetes mellitus (categorical: normal, impaired fasting glucose, and diabetes), hypercholesterolemia (categorical, yes or no), hypertension (categorical, normal, prehypertensive, and hypertensive) and hypertriglyceridemia (categorical, yes or no), Oral contraceptive (yes or no).

### Statistical analysis

Only age was used as a continuous variable; the remaining variables were used as categorical variables. The descriptive characteristics of all variables are presented as unweighted values, complex sample-based weighted percentages (%), and 95% confidence intervals (CIs). The association between breastfeeding duration and periodontitis was tested using logistic regression analysis in a complex sample. Potential confounders or incremental mediators were adjusted for and tested using an adjusted model. Model 1 is adjusted for age. Model 2 was further adjusted for socioeconomic and educational characteristics. Model 3 was additionally adjusted for personal healthcare practice factors. Model 4 was adjusted for systemic medical variables. The statistical model for periodontitis was determined by referring to previous studies [29–32]. After adjusting for multiple variables, the association between breastfeeding duration and periodontitis was determined. Finally, a complex-sample logistic regression model was used to estimate the relationship between breastfeeding and periodontitis in each age group. For all statistical tests, *P* < 0.05 denoted statistical significance. All statistical analyses were performed using the IBM SPSS Statistics version 26 (IBM Corp., Armonk, NY, USA).

## Results

A total of 5,806 women that participated in the survey were aged ≥ 30 and gave birth in the past. A total of 219 participants had no periodontal status information, and 5,587 parous women were analyzed. Table 1 shows the descriptive characteristics of the study population, including demographics, individual health practices, and medical status. The mean weighted age of the participants was 60.87 years. The participants without periodontitis had a mean weighted age of 53.03 years. Furthermore, approximately three-fifths of women breastfed for more than 12 months. The least number of women had no breastfeeding experience.

**Table 1:** Socio-demographic characterization of the study population according to periodontal status

Variable	Periodontitis				
	Yes		No		*P*-value
	Unweighted N	Weighted % (95% CI)	Unweighted N	Weighted % (95% CI)	
Age, mean (95% ci)	1671	60.87 (60.16–61.58)	3916	53.03 (52.35–53.71)	$<$ 0.001
Breast Feeding					
None	214	14.4 (12.3–16.7)	555	14.2 (13.0–15.5)	$<$ 0.001
1–11 month	254	15.2 (13.3–17.3)	961	24.9 (23.2–26.8)	
≥ 12 month	1169	70.4 (67.5–73.1)	2369	60.8 (58.8–62.9)	
Household income					
Lowest	500	29.2 (26.2–32.4)	695	16.8 (15.1–18.7)	$<$ 0.001
Middle low	464	27.5 (25.3–29.1)	953	24.3 (22.5–26.1)	
Middle high	377	23.2(20.8–25.8)	1110	28.3 (26.6–30.0)	
Highest	326	20.1 (17.4–23.0)	1152	30.6(28.1–33.3)	
Education					
≥ Elemental school	652	37.2 (34.0–40.4)	769	18.7 (17.0–20.5)	$<$ 0.001
Middle school	445	16.7 (14.5–19.1)	445	10.9 (9.6–12.2)	
High school	1167	29.2 (26.4–32.1)	1167	31.8 (30.0–33.8)	
≤ University or college	1533	17.0 (14.7–19.6)	1533	38.6 (36.1–41.1)	
Floss					
No	1409	84.1 (81.7–86.3)	2634	67.1 (65.1–68.9)	$<$ 0.001
Yes	257	15.9 (13.7–18.3)	1270	32.9 (31.1–34.9)	
Interdental brush					
No	1422	86.8 (84.8–88.5)	3074	78.5 (76.9–80.0)	$<$ 0.001
Yes	244	13.2 (11.5–15.2)	830	21.5 (20.0–23.1)	
Daily Toothbrushing					
0–1 time	150	9.2 (7.7–10.9)	208	4.8 (4.0–5.6)	$<$ 0.001
twice	712	42.3 (39.5–45.1)	1452	36.9 (34.7–38.4)	
≥ 3 times	809	48.5 (45.5–51.6)	2256	58.7 (56.7–60.6)	
Self-rated oral health					
Poor	1299	57.7 (54.7–60.6)	962	33.0 (30.9–35.2)	$<$ 0.001
Good	2615	42.3 (39.4–45.3)	708	67.0 (64.8–69.1)	
Smoking					
Never	1510	90.6 (88.7–92.2)	3564	91.4 (90.2–92.4)	$<$ 0.001
Past	63	3.3 (2.5–4.3)	216	5.2 (4.5–6.1)	
Current	93	6.1 (4.7–7.8)	124	3.4 (2.7–4.2)	
Alcohol Drinking					
Never	346	27.6 (24.4–31.0)	808	24.4 (22.6–26.2)	0.076
Current	929	72.4 (69.0–75.6)	2478	75.6 (73.8–77.4)	
Hypercholesterinemia					
No	1067	67.4 (64.8–69.8)	2761	74.2 (72.5–75.8)	$<$ 0.001
Yes	506	32.6 (30.2–35.2)	940	25.8 (24.2–27.5)	
Hypertriglyceridemia					
No	1191	85.7 (83.3–87.9)	2880	91.0 (89.7–92.2)	$<$ 0.001
Yes	181	14.3 (12.2–16.7)	300	9.0 (7.8–10.3)	
Hypertension					
Normal	534	32.5 (29.8–35.3)	2014	52.0 (49.8–54.2)	$<$ 0.001
Prehypertension	369	23 (20.6–25.6)	808	21.2 (19.7–22.9)	
Hypertension	767	44.5 (41.6–47.4)	1091	26.7 (24.8–28.8)	
Diabetes Mellitus					
Normal	857	56.0 (53.1–58.9)	2587	69.7 (68.0–71.4)	$<$ 0.001
Impaired fasting glucose	415	25.3 (22.9–27.8)	765	21.4 (20.0–22.9)	
Diabetes	301	18.7 (16.3–21.3)	343	8.9 (7.8–10.1)	
Oral contraceptive					
No	1305	77.9(75.4–80.2)	3232	83.2(81.7–84.5)	< 0.001
Yes	364	22.1(19.8–24.6)	678	16.8(15.5–18.3)	

*CI* Confidence interval

Age: ≥ 30 years

Sex: Parous women

Table 2 shows the multivariate association between breastfeeding and periodontitis. In all statistical models, there was a 60% higher prevalence of periodontitis in women that did not breast feed compared with women that breastfed for 12 months or longer (model 1, aOR, 1.259; 95% CIs, 1.012–1.567; model 4, aOR, 1.597; 95% CIs, 1.199–2.129).

**Table 2:** Analysis of the association between breastfeeding and periodontitis

	Odds ratio (95% confidence interval)			
	Model 1	Model 2	Model 3	Model 4
Breast feeding duration	*N* = 5,522	*N* = 5,511	*N *= 5,494	*N* = 3,681
none	**1.259 (1.012–1.567)**	**1.418 (1.127–1.782)**	**1.402 (1.108–1.774)**	**1.597 (1.199–2.129)**
1–11 Month	0.905 (0.749–1.094)	1.038 (0.854–1.262)	1.056 (0.866–1.287)	1.159 (0.915–1.469)
≥ 12 Month	Reference	Reference	Reference	Reference

Model 1 was adjusted to social status variables (age)

Model 2 was additionally adjusted for sociodemographic variables (household income and education levels)

Model 3 was additionally adjusted for personal healthcare practice variables (dental flossing, interdental brushing, daily tooth brushing, and self-rated oral health)

Model 4 was additionally adjusted for medical status variables (smoking status, alcohol consumption, diabetes mellitus, hypercholesterolemia, hypertension, hypertriglyceridemia and oral contraceptive)

Bold denotes ~ *P* < 0.05

Table 3 shows the results of the model of multiple associations between breastfeeding and periodontitis according to age group. The difference was statistically significant only in the 50–59 age group (*P* < 0.05). Women aged 50–59 years who had never breastfed were more likely to develop periodontitis than those who had breastfed for ≥ 12 months. (aOR, 1.678; 95% CIs, 1.046–2.691).

**Table 3:** Analysis of the association between breastfeeding and periodontitis in different age groups

Periodontitis	Duration of Breast feeding		
	Over 12 Months	1–11 Months	None
Age			
30–39	*N* = 111	*N* = 335	*N* = 398
aOR (95% CI)	Reference	2.044 (0.999–4.018)	1.108 (0.379–3.237)
40–49	*N* = 259	*N* = 421	*N* = 531
aOR (95% CI)	Reference	0.944 (0.574–1.551)	1.312 (0.760–2.262)
50–59	*N* = 254	*N* = 317	*N* = 765
aOR (95% CI)	Reference	1.344 (0.876–2.063)	**1.678 (1.046–2.691)**
60–69	*N* = 95	*N* = 120	*N* = 908
aOR (95% CI)	Reference	0.838 (0.459–1.528)	1.125 (0.535–2.366)
≥ 70	*N* = 50	*N* = 22	*N* = 936
aOR (95% CI)	Reference	0.627 (0.179–2.194)	1.915 (0.677–5.418)

Confounder variables: household income level, education level, use of dental floss, use of interdental brushes, daily brushing frequency, self-related oral health, smoking, alcohol consumption, diabetes mellitus, hypertension, hypercholesterolemia, and hypertriglyceridemia, oral contraceptive

*CI* Confidence interval, *aOR* Adjusted odds ratio

Bold denotes ~ *P* < 0.05

## Discussion

This study investigated the association between breastfeeding duration and periodontitis. The results showed that women who breastfed for more than 12 months had a lower risk of periodontitis than non-breastfeeding women, suggests that breastfeeding may have a protective effect on periodontal health. This finding is not consistent with previous researches, which found that a longer duration of breastfeeding could significantly increase periodontitis in postmenopausal Korean women [24]. One possible explanation for this result is that postmenopausal women typically have a higher risk of periodontitis due to hormonal changes and other factors. As described in introduction, the reference group of breastfeeding is different from our study. The previous study could match the elderly who give birth to many children, and this could have bias, which is disadvantageous for periodontal disease research. On the other hand, our study used the reference group that did not breastfeeding as a reference, and other groups are 1–11 months and 12 months or more. It is assumed that the results are naturally different from this previous study and can be reasonable considering low birthrate in Korea. Another previous study revealed that the prevalence rate of periodontal disease was 6.915 times higher when the length of the lactation period was 14 years in Korean female adults [25]. However, the study only analyzed data for one year and did not consider personal healthcare practice, and systematic medical factors. Sample size and whether to consider these variables that directly associated with periodontal health may be the reason for our inconsistent results. Nevertheless, the findings from studies suggest that breastfeeding may have a significant impact on periodontal health, and should be considered as an important factor in promoting oral health and preventing periodontitis in women. It should be noted that the correlation between breastfeeding and periodontitis is multifaceted and can be impacted by several factors., more research is needed to better understand the mechanisms underlying these effects. The mechanism underlying the association between breastfeeding and periodontitis is not yet clear, but there are several hypothesized ways linking the two.

One of the potential mechanisms is related to common risk factor. Periodontitis is a disease characterized as a noncommunicable disease (NCD), is known to be associated with breast cancer, cardiovascular disease (CVD), diabetes, hypertension, Alzheimer's disease, and depression [33]. In that regard, Breastfeeding may reduce the risk of breast cancer, and a significant association between periodontitis and breast cancer has been observed [34–38]. This suggests that breastfeeding may indirectly reduce the risk of periodontitis by lowering the risk of breast cancer. Additionally, breastfeeding may reduce the risk of cardiovascular diseases and other related risk factors such as type 2 diabetes, hypertension, and Alzheimer's disease, which have been found to be a risk factor for the onset or progression of periodontitis [39–44]. Another potential mechanism is related to autoimmune diseases, such as rheumatoid arthritis, which may increase the risk of periodontitis [45, 46]. Women who breastfeed for a longer duration may reduce the risk of periodontitis by reducing the risk of autoimmune disease [47, 48]. Finally, depression may also be a factor, as non-breastfeeding women have been found to be more depressed and anxious than breastfeeding women [49–52]. Depression has been linked to an increased risk of periodontitis [53, 54], and therefore, breastfeeding may indirectly lower the risk of periodontitis by reducing the incidence of depression.

Overall, the study suggests that breastfeeding may have a protective effect against periodontitis, but the mechanisms underlying this association require further investigation. Additionally, the potential links between breastfeeding and breast cancer, cardiovascular disease, autoimmune disease, and depression should be explored in more detail to better understand the relationship between breastfeeding and periodontitis. It is necessary to analyze this part with the integrated data by period of the Korean National Health and Nutrition Examination Survey as a further study.

This study has some limitations. Although it was a National Health Survey, national surveys have several limitations. Periodontitis was assessed using the CPI, which may have underestimated or overestimated outcome [55]. This cross-sectional study does not support a causal relationship between breastfeeding and the prevalence of periodontitis. Additionally, participation in the questionnaire was subject to memory bias and may have contained some inaccuracies. Potential bias could not be ruled out because missing periodontal status data were excluded. Finally, since the study population consisted of Korean women, it is difficult to apply the results to all populations, and a different correlation pattern was observed in other populations.

Despite these limitations, this study has several advantages. First, this is the first study to report that a longer breastfeeding duration may reduce the risk of periodontitis. Second, we used a large national dataset and analyzed a sufficient number of samples. Our findings provide support for maternal oral health education. However, to the best of our knowledge, no studies to date have directly shown that longer breastfeeding duration reduces the risk of periodontitis. Therefore, one important future direction for this study is to thoroughly investigate the precise mechanism underlying the association between breastfeeding and periodontitis.

## Conclusion

From the above discussion, it can be concluded that longer breastfeeding duration is significantly associated with a reduced risk of periodontitis in Korean women. This study suggests that breastfeeding may have a protective effect on periodontal health in women. This finding adds another benefit to women who choose to breastfeed. To strengthen the periodontal health management of women, our results can provide literature support for the future oral health education of lactating women.

## Data Availability

The data used in this study were obtained from the 7th KNHANES (2016–2018) conducted by the KCDCP. The data that support the findings of this study are available on request from the corresponding author, Hyun-Jae Cho. Access to the data can be obtained by visiting the following website and completing the application [https://knhanes.kdca.go.kr/knhanes/sub03/sub03_02_05.do].

## Abbreviations

KNHANES: Korea National Health and Nutrition Examination Survey
KCDCP: Korea Centers for Disease Control and Prevention
CIs: Confidence Intervals
OR: Odds Ratio
CPI: Community Periodontal Index
KDCA: Korea Disease Control and Prevention Agency
